# Ionic Mechanisms Underlying Bistability in Spinal Motoneurons: Insights from a Computational Model

**DOI:** 10.1101/2025.06.06.658369

**Published:** 2025-06-10

**Authors:** Yaroslav Molkov, Florent Krust, Russell Jeter, Tommy Stell, Mohammed A. Y. Mohammed, Frédéric Brocard, Ilya A. Rybak

**Affiliations:** 1 Department of Mathematics and Statistics, Georgia State University, Atlanta, GA, USA; 2 Neuroscience Institute, Georgia State University, Atlanta, GA, USA; 3 Institut de Neurosciences de la Timone, Aix Marseille University, CNRS, Marseille, France; 4 Department of Neurobiology and Anatomy, Drexel University College of Medicine, Philadelphia, PA, USA

## Abstract

Spinal motoneurons are the final output of spinal circuits that engage skeletal muscles to generate motor behaviors. Many motoneurons exhibit bistable behavior, alternating between a quiescent resting state and a self-sustained firing mode. This bistability is traditionally attributed to plateau potentials, which are driven by persistent inward currents. This intrinsic property is important for normal movement control, but can become dysregulated, causing motor control deficits, like spasticity. Here, we use a conductance-based single-compartment model to investigate the ionic conductances underlying the bistable behaviour of motoneurons. Our simulations demonstrate that the motoneuron bistability and how its emergence is regulated mainly depends on the interplay between several intrinsic ionic mechanisms. In particular, the calcium-activated nonspecific cation current $\left(I_{CAN}\right)$, which is amplified by $I_{CaL}$ and calcium-induced calcium release (CICR), primarily drives the plateau potential to sustain bistability. Additional modulation is provided by the persistent sodium current $\left(I_{NaP}\right)$ and the calcium-dependent potassium current $\left(I_{KCa}\right)$. This study provides a mechanistic model of motoneuron bistability, offering insights into its disruption in pathological conditions and setting the stage for future research and therapeutic development.

## Introduction

Motoneurons show a variety of nonlinear intrinsic behaviors that determine their input-output properties (Binder et al., 2020). Among them, bistability allows motoneurons to toggle between two stable states: a quiescent resting state, and a self-sustained firing state consisting of regular pattern of action potentials in absence of any synaptic drive. A short excitatory input can induce the transition from the quiescent state to the active state. Conversely, a short inhibitory input can induce the opposite transition (Hultborn et al., 1975; Hounsgaard et al., 1984). The underlying mechanism of the bistability involves plateau potentials, long-lasting membrane depolarizations during which the discharge is maintained (Hounsgaard, Hultborn, et al., 1988; Hounsgaard & Mintz, 1988). Persistent firing described in motor units in intact animals (Eken et al., 1989; Eken & Kiehn, 1989; Kiehn & Eken, 1997) as well as in humans (M. A. Gorassini et al., 1998; M. Gorassini et al., 1999; Collins et al., 2001) provide indirect evidence that intrinsic bistability is of functional importance during behavior. It has been suggested to support postural control by reducing the amount of constant synaptic input needed (Hounsgaard, Hultborn, et al., 1988; Lee & Heckman, 1998a; Heckman et al., 2009). Consistent with this, bistability is thought to exist in low-threshold motoneurons (Lee & Heckman, 1998b) and it has recently been found that limiting bistability results in postural deficits (Bos et al., 2021).

Early studies proposed that motoneuron bistability was mainly due to a persistent component of the calcium current $\left(I_{CaL}\right)$ (Hounsgaard & Mintz, 1988; Hounsgaard & Kiehn, 1989) conducted through Cav1.3 channels (Carlin et al., 2000; Simon et al., 2003; Zhang et al., 2006). However, subsequent research has shown that bistability results from a more complex interaction of several ionic currents (Bouhadfane et al., 2013) that includes a calcium-activated nonspecific cation current $\left(I_{CAN}\right)$ flowing through TRPM5 channels (Bos et al., 2021) and the persistent sodium current $\left(I_{NaP}\right)$ via Nav1.6 channels (Drouillas et al., 2023). The emerging view is that membrane depolarization activates $I_{NaP}$, triggering firing and calcium influx. $I_{CaL}$ activation increases intracellular calcium, which then activates $I_{CAN}$, further depolarizing the membrane. This cascade of depolarization and triggered currents leads to self-sustained firing, reinforcing the positive feedback loop underlying bistability. However, the contribution of these intrinsic processes in motoneuron bistability is closely tied to cell size with larger motoneurons exhibiting both higher current expression and a greater propensity for bistable behavior (Harris-Warrick et al., 2024).

Bistability can be modulated by several external factors including monoamines released from supraspinal centers. These neuromodulatory signals are particularly effective in promoting bistability in motoneurons with latent or partial bistable properties (Conway et al., 1988; Hounsgaard, Hultborn, et al., 1988; Hounsgaard & Kiehn, 1989; Perrier & Delgado-Lezama, 2005). In addition, temperature also modulates bistability: values above 30°C unmask plateau potentials by recruiting thermosensitive currents mediated by TRPM5 channels (Bos et al., 2021).

Beyond its physiological role, bistability has critical implications in pathological contexts. Following spinal cord injury, excessive bistability in motoneurones has been linked to spasticity, a disabling neurological condition characterized by involuntary muscle contractions (Bennett et al., 2001; Heckman et al., 2008). This hyperexcitability arises from dysregulated ionic currents that amplify self-sustained firing and disrupt motor control (C. Brocard et al., 2016; Murray et al., 2010).

Despite significant progress, our understanding of the complex regulation of motoneuron intrinsic dynamics, particularly the integration of multiple ionic mechanisms underlying bistability, remains incomplete. Computational models addressing single ionic currents in isolation and not their interactions have been frequently applied in the past, leaving open important questions about their combined effects on motoneuron excitability. Closing these gaps is essential not only for advancing basic neuroscience but also to predict how altered ionic conductances contribute to pathological states. In this study, we use a computational model that combines key ionic conductances into a unified motoneuron model. The purpose is to dissect how these currents, separately and combined, contribute to the emergence and regulation of bistability in motoneurons.

## Methods

### Modeling methods

#### Motoneuron model

We used a conductance-based single-compartment mathematical model of a motoneuron that includes the main spike-generating channels, fast sodium $\left(I_{NaF}\right)$ and potassium rectifier $\left(I_{Kdr}\right)$, as well as the persistent sodium $\left(I_{NaP}\right)$, slowly inactivating potassium $I_{Kv1.2})$, high-voltage activated calcium $\left(I_{CaL}\right)$, $Ca^{2+}$-activated, cation non-specific ($I_{CAN}$, associated with TRPM5 channels) and $Ca^{2+}$--dependent potassium ($I_{KCa}$, associated with SK channels) current.

The voltage dynamics are described by the current balance equation:

(1) $$C\cdot \frac{dV}{dt}=-I_{NaF}-I_{NaP}-I_{Kdr}-I_{Kv1.2}-I_{CaL}-I_{KCa}-I_{CAN}-I_{L}+I_{inj},$$

where V is membrane potential in mV, t is time in ms, C is membrane capacitance $\left(C=1\;\mu F\cdot cm^{-2}\right)$, and the right hand side of the equation contains all transmembrane currents described as follows.

Fast sodium current (Booth et al., 1997):

$$I_{NaF}=g_{NaF}\cdot m_{NaF}{\left(V\right)}^{3}\cdot h_{NaF}(V)\cdot (V-E_{Na});\;m_{NaF}(V)={\left(1+\text{exp}(-\;\left(V+35\right)∕7.8)\right)}^{-1};\;\tau_{NaF}(V)\cdot \frac{dh_{NaF}}{dt}=h_{NaF^{\infty }}(V)-h_{NaF};\;\tau_{NaF}(V)=30\cdot {\left(\text{exp}(\left(V+50\right)∕15)+\text{exp}(-\;\left(V+50\right)∕16)\right)}^{-1};\;h_{NaF\infty }(V)={\left(1+\text{exp}(\left(V+55\right)∕7)\right)}^{-1},\;g_{NaF}=120\;mS∕cm^{2},\;E_{Na}=55\;mV.$$

The persistent sodium current was assumed to be non-inactivating. Activation was assumed instantaneous and non-inactivating. Activation dependence on voltage was taken from (F. Brocard et al., 2013), :

$$I_{NaP}=g_{NaP}\cdot m_{NaP}(V)\cdot h_{NaP}(V)\cdot (V-E_{Na});\;m_{NaP}(V)={\left(1+\text{exp}(-\;\left(V+53\right)∕3)\right)}^{-1};$$

Persistent sodium current is assumed non-inactivating, i.e. $h_{NaP}(V)=1$.

Potassium rectifier current (Booth et al., 1997):

$$I_{Kdr}=g_{Kdr}\cdot m_{Kdr}{\left(V\right)}^{4}\cdot (V-E_{K});\;\tau_{Kdr}(V)\cdot \frac{dm_{Kdr}}{dt}=m_{Kdr^{\infty }}(V)-m_{Kdr};\;\tau_{Kdr}(V)=7\cdot {\left(\text{exp}(\left(V+40\right)∕40)+\text{exp}(-\;\left(V+40\right)∕50)\right)}^{-1};\;m_{Kdr\infty }(V)={\left(1+\text{exp}(\;-\left(V+28\right)∕15)\right)}^{-1};\;g_{Kdr}=100\;mS∕cm^{2},\;E_{K}=26.54\cdot \log(K_{out}∕K_{in}),\;K_{in}=140\;mM,\;K_{out}\in [4,12]\;mM.$$

Slowly inactivating potassium current (Bos et al., 2018):

$$I_{Kv1.2}=g_{Kv1.2}\cdot m_{Kv1.2}(V)\cdot h_{Kv1.2}(V)\cdot (V-E_{K});\;\tau_{Kv1.2}^{x}(V)\cdot \frac{dx_{Kv1.2}}{dt}=x_{Kv1.2\infty }(V)-x_{Kv1.2},\;x\in \{m,\;h\};\;\tau_{Kv1.2}^{m}(V)=2.44+18.\;387\cdot {\left(\text{exp}(-\;\left(V-25.645\right)∕21.633)+\text{exp}(\left(V+4.42\right)∕45.9)\right)}^{-1};\;m_{Kv1.2\infty }(V)={\left(1+\text{exp}(-\;\left(V+46\right)∕6.9)\right)}^{-1};\;\tau_{Kv1.2}^{h}(V)=74.74\cdot {\left(0.\;00015\cdot \text{exp}(-\;\left(V+13\right)∕15)+0.\;06\cdot {\left(1+\text{exp}(-\;\left(V+68\right)∕12)\right)}^{-1}\right)}^{-1};\;h_{Kv1.2\infty }(V)={\left(1+\text{exp}\left((V+54\right)∕7.1)\right)}^{-1};\;g_{Kv1.2}\in [0,10]\;mM.$$

High-voltage calcium $L\;(I_{CaL})$ current (Jasinski et al., 2013):

$$I_{Cal}=g_{CaL}\cdot m_{CaL}(V)\cdot h_{CaL}(V)\cdot (V-E_{Ca});\;\tau_{CaL}^{x}\cdot \frac{dx_{CaL}}{dt}=x_{CaL\infty }(V)-x_{CaL},\;x\in \{m,\;h\};\;m_{CaL\infty }(V)={\left(1+\text{exp}(-\;\left(V+27.5\right)∕5.7)\right)}^{-1};\;h_{CaL\infty }(V)={\left(1+\text{exp}(\left(V+52.4\right)∕5.2)\right)}^{-1};\;E_{Ca}=80\;mV,\;g_{CaL}=0.\;05\;mS∕cm^{2},\;\tau_{CaL}^{m}=0.\;5\;ms,\;\tau_{CaL}^{h}=18\;ms.$$

Calcium-activated High-voltage calcium cation nonspecific $\left(I_{CAN}\right)$ current (Toporikova & Butera, 2011):

$$I_{CAN}=g_{CAN}\cdot \frac{Ca}{Ca+K_{CAN}}\cdot (V-E_{CAN}),\;K_{CAN}=0.\;74\cdot 10^{-3}\;mM,\;E_{CAN}=0\;mV.$$

Calcium-dependent potassium current $\left(I_{KCa}\right)$ (Booth et al., 1997). This current was modified to be instantaneous:

$$I_{KCa}=g_{KCa}\cdot \frac{Ca}{Ca+K_{d}}\cdot (V-E_{K}),\;K_{d}=0.2\cdot 10^{-3}\;mM.$$

Leak current:

$$I_{L}=g_{L}\cdot (V-E_{L}),\;g_{L}=0.1\;mS∕cm^{2},\;E_{L}=-80\;mV.$$

The two currents described above ($I_{CAN}$ and $I_{KCa}$) depend on the intracellular $Ca^{2+}$ concentration (Ca). The $Ca^{2+}$ concentration increases directly from the influx of calcium ions through calcium channels (captured by $I_{CaL}$ in the mathematical model) and indirectly from the release of calcium ions from intracellular stores via a calcium-induced calcium release (CICR) mechanism. Additionally, they are pumped out by the Ca-ATP pumps.

The dynamics of intracellular calcium concentration (Ca) in our model are described by the differential equation:

(2) $$\frac{dCa}{dt}=-f\cdot \alpha \cdot I_{CaL}+k_{CICR}\cdot Ca-Ca∕\tau_{Ca}.$$

On the right hand side of this equation, the first term represents calcium influx through high-threshold, voltage-gated calcium channels $\left(I_{CaL}\right)$ which open during action potentials. Here f=0.01 defines the ratio of entered Ca^2+^ ions remaining unbound; the coefficient $\alpha ={\left(2\cdot F\cdot \delta \right)}^{-1}$ converts inward $I_{CaL}$ current to $Ca^{2+}$ concentration rate of change; here F is Faraday’s constant $\left(F=9.648\cdot 10^{4}\;C∕mol\right)$ and δ is the thickness (0.1μm) of the shell adjacent to the membrane. Based on these parameters, $\alpha =5\cdot 10^{-4}\;mM\cdot cm^{2}\cdot ms^{-1}\cdot \mu A^{-1}$.

The second term describes an increase of cytoplasmic $Ca^{2+}$ through the CICR mechanism, where the rate of calcium release from internal stores is proportional to the intracellular $Ca^{2+}$ concentration (defined by $k_{CICR}$). The third term describes the action of calcium pumps (both plasma membrane and SERCA) which rapidly remove calcium from the cytoplasm, with a time constant $\tau_{Ca}=10\;ms$.

#### Qualitative analysis

To investigate the bistable behavior of spinal motoneurons, we implemented a current ramp simulation protocol designed to probe the transitions between quiescent and self-sustained firing states. This approach leverages a linearly varying injected current $\left(I_{inj}\right)$ to systematically explore the system’s response across a range of input intensities, making it an effective tool for detecting hysteresis and state-dependent dynamics in neuronal models.

The protocol was designed as follows: the injected current was initially set to zero and then increased linearly to a predetermined maximum value (ascending phase) over a specified duration. Subsequently, the current was decreased linearly back to zero (descending phase) at the same rate. This bidirectional ramp allowed us to identify two key transition points: the current threshold at which the system shifts from silence to spiking during the ascending phase $\left(I_{up}\right)$ and the lower threshold at which spiking ceases during the descending phase $\left(I_{down}\right)$. Bistability is indicated when $I_{down}$ is less than $I_{up}$, revealing a range of current values where the system can stably maintain either state, depending on its prior condition. This hysteresis reflects the nonlinear properties of the model and its history-dependent behavior. To ensure the reliability of these thresholds, the ramp time was varied systematically, extending the duration of the ascending and descending phases until $I_{up}$ and $I_{down}$ stabilized, confirming that the observed transitions were not influenced by transient dynamics. Typically, ramp durations spanned several hundred milliseconds, with longer durations tested to exclude such effects.

To further validate the protocol’s ability to detect bistability, we employed a complementary current step simulation inspired by experimental approaches. Starting from a baseline of $I_{inj}=0$, the current was stepped to an intermediate value within the suspected bistable range $\left(I_{down}<I_{inj}<I_{up}\right)$, then increased to a level above $I_{up}$, and subsequently returned to the intermediate value before returning to zero. This sequence demonstrated that, at the intermediate current level, the system’s state—silent or spiking—depended on its prior activation history, reinforcing the findings of the ramp protocol.

The current ramp simulations also supported parametric analyses by varying key model parameters and plotting the resulting bifurcation diagrams. These diagrams mapped the system’s equilibrium points and oscillatory regimes as functions of $I_{inj}$, providing a visual representation of the bistable region. The protocol’s design, with its carefully adjusted ramp duration, ensured that the gradual variation of input current effectively captured the boundaries of this region, offering a robust technical framework for studying the conditions under which bistability emerges and persists in the model.

#### Simulations

Simulations were performed using custom-written C++ and Julia software. Integration was performed by the Dormand-Prince 5(4) method using ($Boost\;C^{++}\;Libraries$, ver. 1.86). Source code written in C++ and Julia for the model and examples of the ramping protocols can be found in the Github repository associated with this manuscript (Jeter & Molkov, 2025).

## Experimental methods

### Experimental model

Mice (C57/Bl6 background) were housed under a 12h light/dark cycle with *ad libitum* access to water and food. Room temperature was kept between 21-24°C and between 40-60% relative humidity. All animal care and use were conformed to the French regulations (Décret 2010-118) and approved by the local ethics committee (Comité d’Ethique en Neurosciences INT-Marseille, CE71 Nb A1301404, authorization Nb 2018110819197361).

#### In vitro preparations

For the slice preparation, the lumbar spinal cord was isolated in ice-cold (+4°C) artificial CSF (aCSF) solution composed of the following (in mM): 252 sucrose, 3 KCl, 1.25 KH2PO4, 4 MgSO4, 0.2 CaCl2, 26 NaHCO3, 25 D-glucose, pH 7.4. The lumbar spinal cord was then introduced into a 1% agar solution, quickly cooled, mounted in a vibrating microtome (Leica, VT1000S) and sliced (325 μm) through the L4–5 lumbar segments. Slices were immediately transferred into the holding chamber filled with bubbled (95% O2 and 5% CO2) aCSF solution composed of (in mM): 120 NaCl, 3 KCl, 1.25 NaH2PO4, 1.3 MgSO4, 1.2 CaCl2, 25 NaHCO3, 20 D-glucose, pH 7.4, 30-32°C. After a 30-60 min resting period, individual slices were transferred to a recording chamber continuously perfused with aCSF heated to 32-34°C.

#### In vitro recordings

Whole-cell patch-clamp recordings were performed using a Multiclamp 700B amplifier (Molecular Devices) from L4-L5 motoneurons with the largest soma (>400μm2) located in the lateral ventral horn. Patch electrodes (2-4 MΩ) were pulled from borosilicate glass capillaries (1.5 mm OD, 1.12 mm ID; World Precision Instruments) on a Sutter P-97 puller (Sutter Instruments Company) and filled with an intracellular solution (in mM): 140 K+-gluconate, 5 NaCl, 2 MgCl2, 10 HEPES, 0.5 EGTA, 2 ATP, 0.4 GTP, pH 7.3. Pipette and neuronal capacitive currents were canceled and, after breakthrough, the series resistance was compensated and monitored. Recordings were digitized on-line and filtered at 20 kHz through a Digidata 1550B interface using Clampex 10.7 software (Molecular Devices). All experiments were designed to gather data within a stable period (i.e., at least 2 min after establishing whole-cell access).

#### Data quantification

Electrophysiological data analyses were analyzed off-line with Clampfit 10.7 software (Molecular Devices). For intracellular recordings, several basic criteria were set to ensure optimum quality of intracellular recordings. Only cells exhibiting a stable resting membrane potential, access resistance (no > 20% variation) and an action potential amplitude larger than 40 mV under normal aCSF were considered. Passive membrane properties of cells were measured by determining from the holding potential the largest voltage deflections induced by small current pulses that avoided activation of voltage-sensitive currents. We determined input resistance by the slope of linear fits to voltage responses evoked by small positive and negative current injections. The peak amplitude of the slow afterdepolarization (slow ADP or sADP) was defined as the difference between the holding potential and the peak voltage deflection after the burst of spikes. The sADP area was measured between the end of the stimulus pulse and the onset of the hyperpolarizing pulse (delta= 7.5 s). In addition, the duration of the sADP was quantified as the time interval at half-maximal amplitude. If necessary, using bias currents, the pre-pulse membrane potential was maintained at the holding potential fixed in the control condition.

Bistable properties were investigated with a 2 s depolarizing current pulses of varying amplitudes (0.8 - 2 nA). To quantify the ability of a motoneuron to be bistable, we gradually increased the holding current in 25 pA steps over a 2-second period before delivering the depolarizing pulse. This process continued until the neuron reached its spiking threshold. The cell was considered as bistable when (1) the pre-stimulus membrane potential stays relatively hyperpolarized below the spiking threshold (downstate), (2) the post-stimulus membrane potential stays depolarized above the spike threshold (upstate), and (3) the membrane potential switches to downstate after a brief hyperpolarizing pulse. To quantify the extent of bistability, we measured both the voltage (V) range between the most hyperpolarized holding potential (Vhmin) and the most depolarized holding potential ((Vhmax) at which the motoneuron can exhibit a self-sustained spiking, and the corresponding range of injected currents (I) over which bistable behavior was observed.

### Statistics

No statistical method was used to predetermine sample size. When two conditions (control vs drugs) were compared, we used the Wilcoxon matched pairs test. For all statistical analyses, the data met the assumptions of the test and the variance between the statistically compared groups was similar. The level of significance was set at p < 0.05. Statistical analyses were performed using Graphpad Prism 7 software.

## Results

### Calcium dynamics and calcium-dependent currents

#### The role of calcium-induced calcium release (CICR) mechanism

A brief excitatory current injected into a motoneuron triggers a series of action potentials, causing a substantial increase in intracellular calcium ($Ca^{2+}$) levels. This increase is driven by influx through voltage-gated calcium channels directly and amplified by the calcium-induced calcium release (CICR) mechanism releasing $Ca^{2+}$ from internal stores. The accumulated intracellular $Ca^{2+}$ activates two major currents: the depolarizing calcium-activated nonspecific cation current $\left(I_{CAN}\right)$ and the hyperpolarizing calcium-dependent potassium current $\left(I_{KCa}\right)$.

As described in the Methods section, the dynamics of intracellular $Ca^{2+}$ in our model are governed by the differential equation (2). The calcium clearance pump operates with a time constant of $\tau_{Ca}=10\;\mathrm{ms}$, which, in the absence of CICR, would clear all calcium introduced by a spike before the next spike. However, the term $k_{CIRC}\cdot Ca$ captures the CICR mechanism, where the rate of calcium release from internal stores is directly proportional to the current calcium concentration Ca with coefficient $k_{CICR}$.

For simplicity, the intracellular calcium dynamics can be expressed as:

$$\frac{dCa}{dt}=-f\cdot \alpha \cdot I_{CaL}-Ca∕\tau_{eff},$$

where

$$\tau_{eff}={\left(1∕\tau_{Ca}-k_{CICR}\right)}^{-1}.$$

The gain $k_{CIRC}$ must not exceed $1∕\tau_{Ca}$ to prevent negative $\tau_{eff}$, which would lead to an infinite increase in calcium. In our model, $k_{CICR}$ is set at 0.096 ms^−1^, yielding an effective time constant of $\tau_{eff}=250\;\mathrm{ms}$.

**Figure 1.**
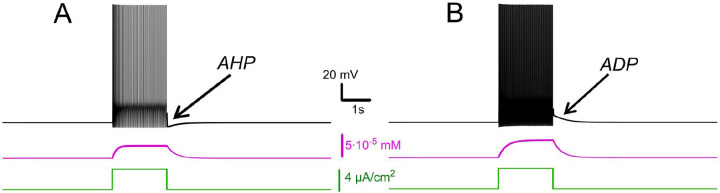
Effects of $I_{KCa}$ vs. effects of $I_{CAN}$. Membrane potential (black) and intracellular $Ca^{2+}$ (magenta) in response to step current (green). Intracellular $Ca^{2+}$ concentration increases during spiking activity and then slowly adapts. **A**. When $I_{KCa}$ is stronger than $I_{CAN}(g_{KCa}=0.5\;\text{mS},g_{CAN}=0\;\text{mS})$, this leads to after-hyperpolarization (AHP). **B**. When $I_{CAN}$ is stronger than $I_{KCa}(g_{KCa}=0.5\;\text{mS},g_{CAN}=0.7\;\text{mS})$, this leads to after-depolarization (ADP) immediately following spiking activity.

This prolonged $\tau_{eff}$ reflects how the CICR mechanism substantially slows calcium clearance, resulting in calcium buildup during repetitive firing. As shown in Figure 1, calcium levels sharply increase in response to a rectangular current pulse, which in turn activates both the calcium-induced cation-nonspecific current $\left(I_{CAN}\right)$ and the calcium-dependent potassium current $\left(I_{KCa}\right)$. Without CICR, calcium would be rapidly cleared by cellular pumps, thus preventing significant accumulation during repetitive firing. Consequently, in the absence of sustained elevations in intracellular $Ca^{2+}$, neither $I_{CAN}$ nor $I_{KCa}$ currents would be activated to a significant degree.

#### Interplay between $I_{CAN}$ and $I_{KCa}$: ADP versus AHP

Physiological experiments have shown that motoneurons demonstrate two types of post-stimulus responses: a bistable motoneuron type expressing a slow afterdepolarization (sADP) after brief excitatory inputs, and a non-bistable motoneuron type expressing an afterhyperpolarization (AHP) (Harris-Warrick et al., 2024). Our model accurately reproduces these phenomena, and links them to motoneuron bistability.

In the model, intracellular calcium levels do not immediately return to baseline after stimulation but decrease gradually over the effective time constant $\tau_{eff}$ (250 ms in our model). The relative contributions of $I_{CAN}$ and $I_{KCa}$ during this phase critically influence neuronal behavior. When $Ca^{2+}$ predominates, an increase of cytosolic $I_{KCa}$ activates potassium efflux that leads to hyperpolarization of the membrane and a decrease in excitability. This effect promotes spike frequency adaptation during firing and leads to a prominent post-stimulus AHP supported by sustained calcium levels and prolonged $I_{KCa}$ activation (Fig. 1A).

Conversely, when $I_{CAN}$ prevails, the elevation of intracellular $Ca^{2+}$ activates this current, mediated by TRPM5 channels, leading to a depolarizing influx of sodium, that in turn increases the firing frequency during stimulation and produces an sADP afterward (Fig. 1B). As calcium drops, the membrane potential slowly relaxes back toward its resting values. These modeled responses are consistent with experimental records of bistable and non-bistable motoneurons.

### Mechanisms of $I_{CAN}$-based bistability and its modulation

#### $I_{CAN}$-based bistability

Our model predicts that the expression of $I_{CAN}$ provides a robust bistability mechanism. The process begins with the opening of voltage-gated calcium channels during each action potential. While this influx alone is insufficient to fully activate $I_{CAN}$, it triggers CICR from intracellular stores, significantly amplifying the cytoplasmic $Ca^{2+}$ concentration levels (see below).

This elevated $Ca^{2+}$ activates $I_{CAN}$ establishing a positive feedback loop: $I_{CAN}$ sustains membrane depolarization, promoting continuous firing, with each spike further promoting CICR and reinforcing $I_{CAN}$ activation. This self-perpetuating mechanism allows the motoneuron to maintain a high-activity state (persistent spiking) even after the initial stimulus is removed, characterizing bistability. The neuron can thus operate in either a quiescent state or an active spiking state.

**Figure 2.**
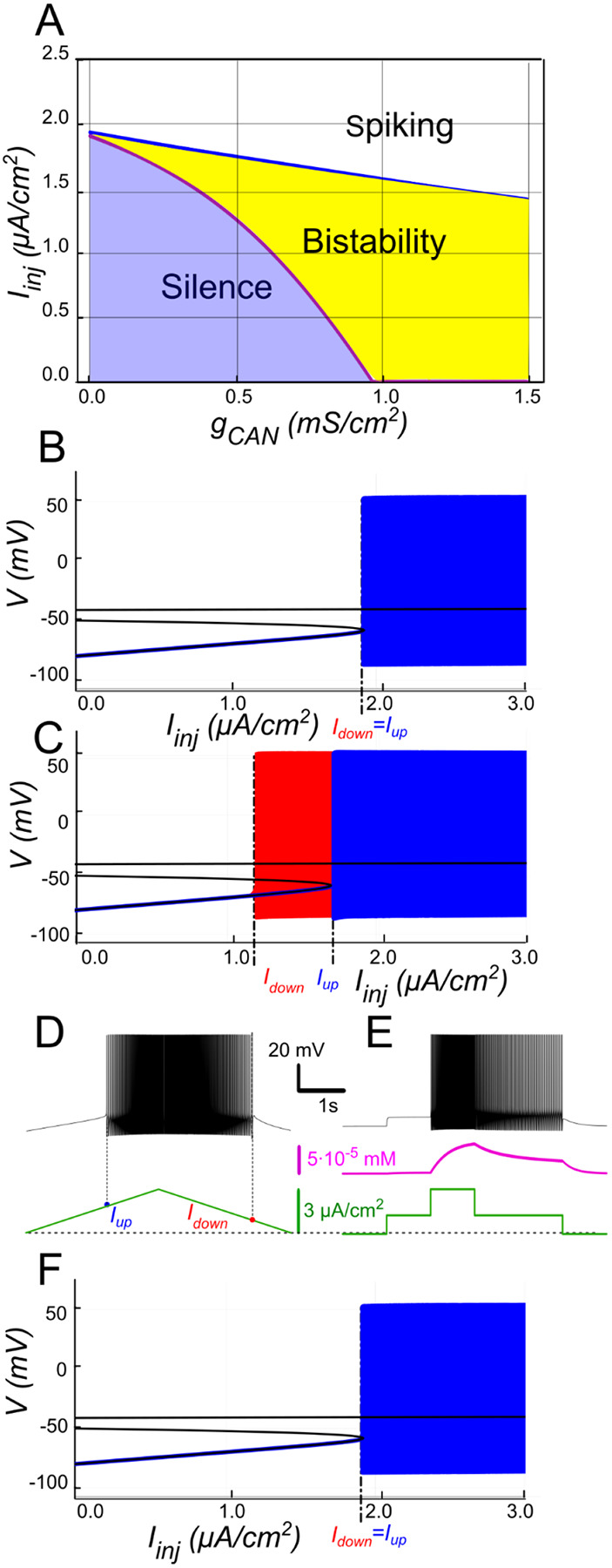
$I_{CAN}$-induced bistability and its disappearance after CIRC blockade. **A:** Parameter plane ($g_{CAN}$, $I_{inj}$) partitioned into regions of different behaviors. The upper and lower boundaries of the bistability region represent the dependence of $I_{up}$ (blue line) and $I_{down}$ (red line) on $g_{CAN}$, respectively. With no $I_{CAN}(g_{CAN}=0)$ the transitions from silence to spiking and back occur at the same $I_{inj}$ values signifying no bistability. As $g_{CAN}$ increases, $I_{down}$ becomes smaller than $I_{up}$, with the bistability range progressively expanding. **B, C**: **Bifurcation diagrams showing different behaviors of the system** at $g_{CAN}=0$ and $g_{CAN}=0.5\;mS∕cm^{2}$ across a range of the injected current values, $I_{inj}$. Black line depicts the system’s equilibrium states. When $I_{inj}<I_{up}$ there is a stable hyperpolarized state (stable node). If $I_{inj}$ is increased over $I_{up}$, the system transitions to spiking, covering the voltage range shown in red and blue. This stable spiking regime exists in the range $I_{inj}>I_{down}$. When $I_{inj}$ is reduced below $I_{down}$, the limit cycle representing spiking disappears, and the system transitions to the low voltage stable fixed point. Between these bifurcation points the hyperpolarized state (silence) coexists with the stable limit cycle (spiking) shown in red. **D, E: Bistability revealed with ramp and step protocols**. **D**: $I_{inj}$ was linearly increased from 0 to 3 $\mu A∕cm^{2}$ and back (green). Note that spiking started at higher current than the transition back to silence $\left(I_{up}>I_{down}\right)$. **E**: $I_{inj}$ was held piecewise constant at 0 first, then increased to $1.5\mu A∕cm^{2}$, then to 3, then reduced back to 1.5, and, finally, to 0 (see the black trace at the bottom). At $I_{inj}=1.5\mu A∕cm^{2}$ the system exhibits spiking or silence depending on whether it was active or not during the previous stage. Note the difference in the intracellular calcium concentration levels (green trace). **F**. Same representation as in C but with intracellular calcium release blocked $\left(k_{CIRC}=0\right)$. Note lack of bistability.

To validate this mechanism, we replicated the experimental ramping current protocol using the mathematical model as described in the “Methods” section. Figure 2C shows the dependence 2 between voltage and linearly varying injected current $I_{inj}$ at $g_{CAN}=0.5\;mS∕cm^{2}$. Below a threshold of $I_{up}=1.7\;\mu A∕cm^{2}$, the system has three equilibrium points: a stable hyperpolarized state corresponding to the silent behavior (stable node), the unstable depolarized state (focus), and a saddle in between (Fig. 2C). As $I_{inj}$ exceeds $I_{up}=1.7\;\mu A∕cm^{2}$, the low potential stable branch of the V-nullcline merges with the saddle and vanishes via a fold bifurcation, and the system transitions to a stable limit cycle representing a repetitive spiking regime. During the descending phase of the ramp, firing persists until $I_{inj}$ decreases to $I_{down}=1.1\;\mu A∕cm^{2}$, which is less than $I_{up}$ (Fig. 2C), revealing a hysteresis indicative of bistability.

To further probe bistability, we implemented a step protocol inspired by experimental methods. Starting at $I_{inj}=0$ in a silent state, we applied an intermediate current within the bistable range $\left(I_{down}<I_{inj}<I_{up}\right)$, then increased it above $I_{up}$ to induce firing, before returning to the intermediate current (Fig. 2D). This protocol showed that, at the same intermediate current value, the motoneuron could either continue firing or remain silent, depending on whether it was previously activated.

The 2-parameter bifurcation diagram in Fig. 2A summarizes how bistability depends on $I_{CAN}$. At $g_{CAN}=0$ the neuron transitions between spiking and silence at the same current threshold during both ascending and descending phases of a ramp protocol, indicative of no hysteresis (Fig. 2B). Once $g_{CAN}$ becomes large enough, bistability emerges. This is evident as the current required to trigger spiking during the ramp-up exceeds the current at which spiking stops during ramp-down (Fig. 2C). This hysteresis widens with further increases in $g_{CAN}$ (Fig. 2A), reflecting an enhanced positive feedback loop driven by $I_{CAN}$, which sustains the self-perpetuating spiking state. These findings underscore the pivotal role of $I_{CAN}$ conductance in modulating the bistable range.

#### The role of CICR

The model demonstrates that voltage-gated calcium channels alone do not allow enough calcium influx to sufficiently raise intracellular calcium levels and activate $I_{CAN}$. To illustrate, intracellular calcium release was blocked by setting $k_{CICR}$ to 0, reducing the value of $\tau_{eff}$ to $\tau_{Ca}=10\;ms$. The result is shown in Fig. 2E. Blocking intracellular calcium release dramatically reduced the transient increase in intracellular calcium concentration. This prevented $I_{CAN}$ activation and disrupted the $I_{CAN}$-based bistability mechanism.

#### The role of $I_{KCa}$ in modulating $I_{CAN}$-based bistability

**Figure 3.**
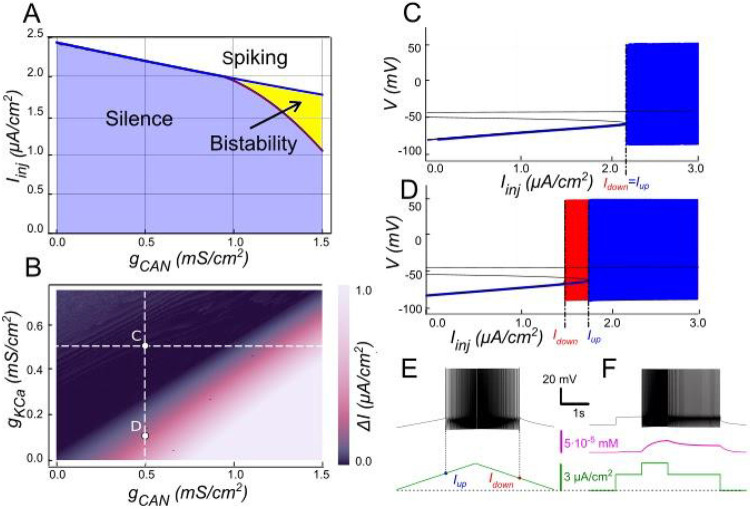
Modulation of $I_{CAN}$-dependent bistability by $I_{KCa}$. **A**. Bifurcation diagram similar to Fig. 2A, constructed for $g_{KCa}=0.5\;mS∕cm^{2}$. Note, that unlike in Fig. 2, bistability emerges once $g_{CAN}$ exceeds $1\;mS∕cm^{2}$. **B**. Bistability range $\Delta I_{inj}$ depending on $g_{CAN}$ and $g_{kCa}$ conductances. Bistability range (defined as $I_{up}-I_{down}$) is color coded. Bistability exists in the lower right part of the diagram. Note near linear dependence of $g_{CAN}$ bistability threshold on $g_{kCa}$. White dashed line shows the value of $g_{kCa}$ used to construct the bifurcation diagram in panel A. **C, D**. Bifurcation diagrams showing possible behaviors of the system at the parameter values labelled correspondingly in panel B. **C**. At higher $g_{kCa}$ value $\left(g_{KCa}=0.5\right)$ transitions from quiescence to spiking and back occur at the same injected current value $\left(I_{up}=I_{down}\right)$, indicating no bistability. **D**. When $g_{kCa}$ is lowered to 0.1, the transition from spiking to quiescence occurs at a lower injected current than the transition from quiescence to spiking $\left(I_{down}<I_{up}\right)$, so spiking shown in red coexists with the silent regime. **E**. Ramp (left) and step (right) current injection protocols, illustrating bistability revealed in D. The intermediate current step is between $I_{down}$ and $I_{up}$. The system’s state depends on whether it was active or not at the previous step, exhibiting bistable behavior.

Our model suggests that $I_{CAN}$-based bistability strongly depends on $I_{KCa}$. When intracellular ${\mathrm{Ca}}^{2+}$ levels increase, $I_{KCa}$ channels open, allowing intracellular potassium $\left(K^{+}\right)$ ions to exit the cell. This outward current hyperpolarizes the membrane, decreasing excitability, and preventing sustained high-activity states. The bifurcation diagrams in Fig. 3 summarize this dependence. At low values of $g_{CAN}$, where $I_{KCa}$ dominantes over $I_{CAN}$, the neuron transitions between spiking and silence at the same current threshold during both ascending and descending phases, with this threshold being subtly influenced by $g_{CAN}$ (Fig. 3A). Increasing $g_{CAN}$ enhances excitability, thereby lowering the threshold current needed for spiking onset.

However, once $g_{CAN}$ reaches approximately 1 $mS∕cm^{2}$ (Fig. 3A), bistability emerges. This is evident as the current required to trigger spiking during the ramp-up exceeds the current at which spiking stops during ramp-down, with the bistability range widening as $g_{CAN}$ increases further. Additionally, the specific $g_{CAN}$ value at which this bifurcation occurs depends linearly on $g_{KCa}$ (Fig. 3B), suggesting that the bistability arises when $I_{CAN}$ begins to dominate over $I_{KCa}$. This also implies that bistable behavior can be induced by lowering $g_{KCa}$ while $g_{CAN}$ is held constant as demonstrated in Figs. 3C-F.

**Figure 4.**
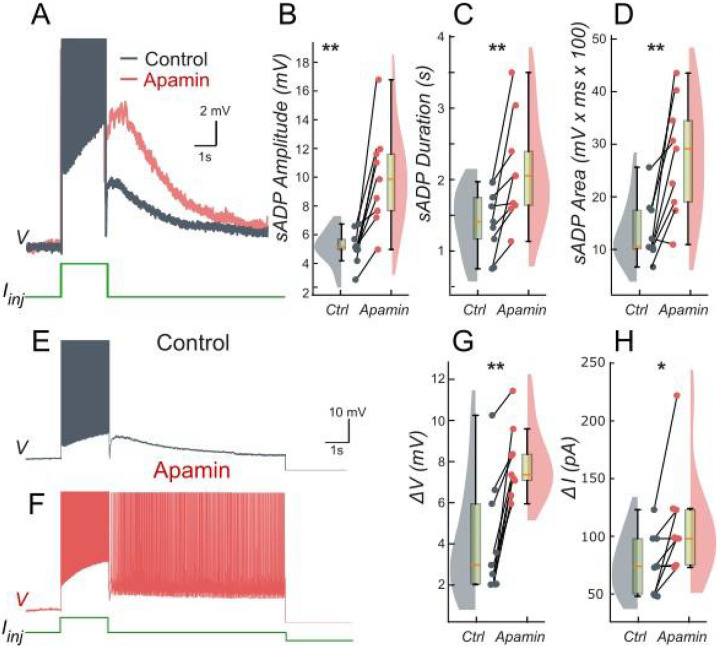
Ca^2+^-activated K+ current $\left(I_{KCa}\right)$ limits the slow afterdepolarization and membrane bistability in lumbar motoneurons. **A**: Superimposed voltage traces recorded in the same motoneuron during a brief (2 s) depolarizing current step (bottom) under control conditions (black) and after bath application of apamin (red). **B-D**: Quantification of the sADP amplitude (**B**), duration(**C**) and area (**D**). **E, F**: Voltage traces in response to a 2-s depolarizing pulse before (**E**) and after apamin (**F**). **G, H:** Quantification of bistability through ΔV and ΔI. ΔV and ΔI represent the range of holding potentials and holding currents, respectively, over which self-sustained firing can be observed. Each is defined as the difference between the most depolarized and the most hyperpolarized value (potential for ΔV, current for ΔI) at which self-sustained firing is triggered or maintained (see Methods). Paired data from individual motoneurons (n = 9) are linked and overlaid on violin and box-and-whisker plots. *P < 0.05, **P < 0.01, two-tailed Wilcoxon signed-rank test.

To further validate these findings, we performed patch-clamp experiments on lumbar motoneurons, focusing on the effects of apamin, a selective blocker of $I_{KCa}$. We measured the parameters of the sADP induced by a brief depolarization of the motoneurons. Application of apamin significantly increased the amplitude, duration, and area of the sADP, indicating a stronger and more prolonged depolarizing response (Fig. 4A-D). In addition, apamin enhanced the capacity of motoneurons for bistable behavior. Specifically, motoneurons were able to generate plateau potentials at more hyperpolarized membrane potentials (Fig. 4E-F), as indicated by a significant shift in Vh min from −62.6 mV to −64.8 mV (p < 0.05; Wilcoxon matched pairs test). This was accompanied by a corresponding increase in both the voltage range (ΔV) and the current range (ΔI) over which bistability was observed (Fig. 4G-H). The increase in ΔV and ΔI reflects the expanded window of membrane potentials and injected currents over which bistability is observed, confirming that reducing $I_{KCa}$ with apamin unmasks $I_{CAN}$-driven bistability by diminishing hyperpolarizing influences, consistent with the prediction of our model (Figs. 1 and 3). These results underscore the antagonistic interplay between $I_{CAN}$ and $I_{KCa}$ in shaping motoneuron excitability and provide a mechanistic basis for the observed post-stimulus bistable behaviors.

#### Modulation of $I_{\text{CAN}}$-based bistability by extracellular potassium concentration

The $I_{KCa}$ plays a pivotal role in modulating $I_{CAN}$-dependent bistability in spinal motoneurons. $I_{KCa}$ is significantly affected by the potassium reversal potential $\left(E_{K}\right)$). This $E_{K}$ value, as defined by the Nernst equation, indicates the potassium ion equilibrium potential based on the ratio of intracellular $\left({K^{+}}_{in}\right)$ to extracellular $\left({K^{+}}_{out}\right)$ potassium concentration (see Methods). When ${K^{+}}_{out}$ increases, $E_{K}$ becomes less negative (shifts toward depolarization), thereby reducing the driving force for potassium efflux through $I_{KCa}$ channels.

This depolarizing shift in $E_{K}$ will decrease the hyperpolarizing effect of $I_{KCa}$ and thus reduce its resistance to the depolarizing impact of $I_{CAN}$. In the bistable mode, $I_{KCa}$ acts as a feedback system that drives the membrane into a hyperpolarized state and prevents stable firing. When in conditions of a low $I_{KCa}$ such as with high ${K^{+}}_{out}$, this balance shifts in favor of $I_{CAN}$. Therefore, the $I_{CAN}$-mediated positive feedback loop is expressed more, which could contribute to the maintained depolarization and prolonged firing activity that we have described above.

**Figure 5.**
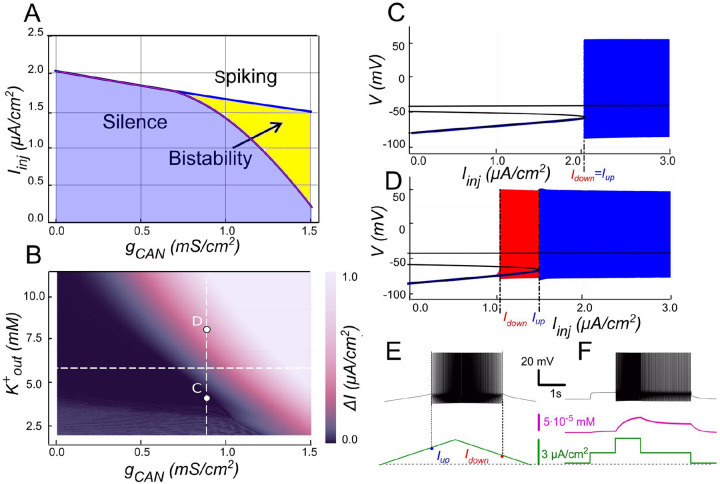
Modulation of $I_{CAN}$-dependent bistability by extracellular potassium concentration $\left({K^{+}}_{out}\right)$. **A**. Bifurcation diagram similar to Fig. 3A, constructed for $g_{KCa}=0.5\;mS∕cm^{2}$, but at elevated $({K^{+}}_{out})=6\mathrm{mM}$ instead of physiologically normal 4mM. Note, that compared to Fig. 3A, bistability emerges at lower $g_{CAN}$. **B**. Color-coded bistability range $\left(I_{up}-I_{down}\right)$ depending on $g_{CAN}$ and $K_{out}$. Black area corresponds to no bistability. White dashed line shows the $\left({K^{+}}_{out}\right)$ value used in panel A. $g_{CAN}$ bifurcation value reduces as $\left({K^{+}}_{out}\right)$ increases, therefore an increase in $\left({K^{+}}_{out}\right)$ can lead to bistability emergence, as shown in **C** and **D**. If $g_{CAN}=0.9\;mS∕cm^{2}$, at $({K^{+}}_{out})=4\;\mathrm{mM}$ (physiologically normal value) no bistability exists (**B**), but if $\left({K^{+}}_{out}\right)$ is raised to 8 mM, bistability emerges (**C**), as illustrated by ramp and step current protocols (**E**).

To explore this interaction, we systematically varied ${K^{+}}_{out}$ and $g_{CAN}$ in our computational model, keeping $g_{KCa}$ fixed at 0.5 mS/cm^2^. Figure 5B illustrates the bistability range as a function of $g_{CAN}$ versus ${K^{+}}_{out}$, with the range of injected current exhibiting bistability color-coded (e.g., black indicating no bistability). At low ${K^{+}}_{out}$ (e.g., physiologically normal levels of 4 mM), $E_{K}$ is strongly negative, and $I_{KCa}$ effectively counteracts $I_{CAN}$, requiring a higher $g_{CAN}$ (e.g., $∼1\;\mathrm{mS}∕{\mathrm{cm}}^{2}$, Fig. 3A) to achieve bistability. As ${K^{+}}_{out}$ increases, $E_{K}$ depolarizes, weakening $I_{KCa}$ and enabling bistability at lower $g_{CAN}$ values (Fig. 5A). For instance, at $g_{CAN}=0.9$ the model switches to bistable behavior as ${K^{+}}_{out}$ is increased from 4 to 8 mM (Fig. 5C, D), with the bistable range widening as ${K^{+}}_{out}$ rises further (Fig. 5B).

This modulation is of physiological relevance, because *in vivo*, during sustained motor activity or pathological conditions, increased ${K^{+}}_{out}$ may promote motoneuron bistability, subsequently affecting motor output. These results demonstrate that intrinsic neuronal properties can be dynamically modulated by environmental factors, such as extracellular ion concentrations, and provide a potential mechanism by which $I_{CAN}$-mediated bistability may be tuned in spinal circuits.

#### The role of $I_{NaP}$ in modulating $I_{CAN}$-based bistability

**Figure 6.**
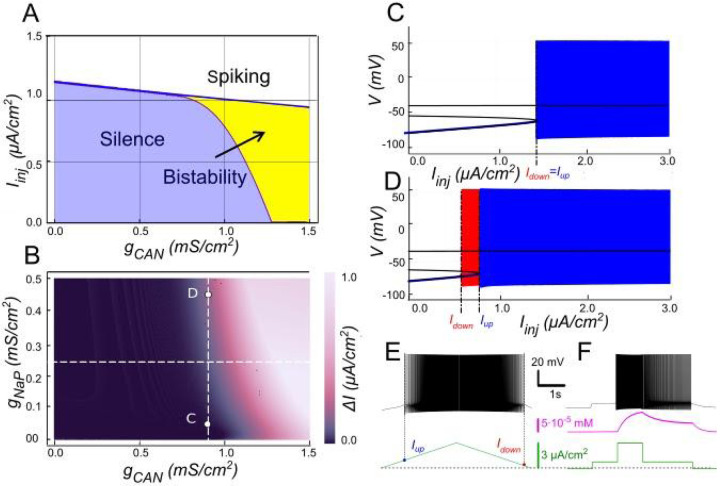
Modulation of $I_{CAN}$-dependent bistability by $I_{NaP}$. **A**. Bifurcation diagram similar to Fig. 3A, constructed for $g_{KCa}=0.5\;mS∕cm^{2}$, but at $g_{NaP}=0.25\;mS∕cm^{2}$ instead of zero. Note, that compared to Fig. 3A, bistability emerges at lower $g_{CAN}$. **B**. Color-coded bistability range $\left(I_{up}-I_{down}\right)$ depending on $g_{CAN}$ and $g_{NaP}$ with $g_{KCa}$ fixed at $0.5\;mS∕cm^{2}$. Black area corresponds to no bistability. $g_{CAN}$ bifurcation value reduces as $g_{NaP}$ increases, therefore an increase in $g_{NaP}$ can lead to bistability emergence, as shown in **C** and **D**. If $g_{CAN}=0.9\;mS∕cm^{2}$, at $g_{NaP}=0$ no bistability exists (**C**), but if $g_{NaP}$ is raised to $0.45\;mS∕cm^{2}$, bistability emerges (**D**), as illustrated by ramp (**E**) and step current protocols (**F**).

$I_{NaP}$ can significantly modulate neuronal activity. We investigated how its expression influences bistability (Fig. 6), revealing a profound impact. Figure 6B illustrates the bistability range (color-coded) across varying $I_{NaP}$ and $I_{CAN}$ conductances ($g_{NaP}$ and $g_{CAN}$, respectively). In this figure, black color indicates no bistability. The bistability range generally expands with increasing $G_{NaP}$. Notably, bistability can emerge even when $I_{CAN}$ alone is insufficient (e.g., $g_{CAN}=0.9\;\text{mS}∕{\text{cm}}^{2}$, $g_{NaP}=0$) by increasing $g_{NaP}$ (Figs. 6C, D).

This phenomenon can be understood by revisiting the mechanism of $I_{CAN}$-induced bistability. Such bistability is enabled by the positive feedback loop: increased neuronal firing elevates intracellular calcium, which activates $I_{CAN}$. The subsequent membrane depolarization drives the firing rate even higher and more calcium influx takes place. The strength of this feedback loop depends on the effectiveness of each step in the process. Enhancing any component of the loop can amplify the gain and promote bistability. In this context, $I_{NaP}$ provides the necessary additional depolarization, thereby amplifying the effect of increased intracellular calcium on firing rate and enabling sustained firing once the neuron is activated. As illustrated, at $g_{CAN}=0.9\;\text{mS}∕{\text{cm}}^{2}$ and $g_{NaP}=0.45\;\text{mS}∕{\text{cm}}^{2}$, depolarization provided by $I_{NaP}$ induces bistability which is not present at $g_{NaP}=0$. At $g_{CAN}=0.5\;\text{mS}∕{\text{cm}}^{2}$, however, the depolarization generated is not sufficient to drive the feedback gain into a positive range.

**Figure 7.**
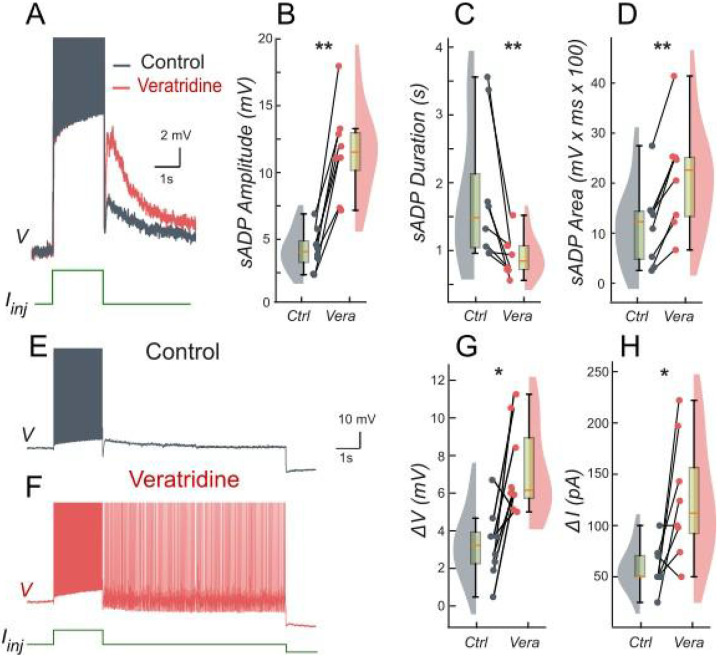
Persistent Na^+^ current $\left(I_{NaP}\right)$ facilitates the slow afterdepolarization and membrane bistability in lumbar motoneurons. **A**: Superimposed voltage traces recorded in the same motoneuron during a brief (2 s) depolarizing current step (bottom) under control conditions (black) and after bath application of veratridine (red). **B-D**: Quantification of the sADP amplitude (**B**), duration (**C**) and area (**D**). **E, F**: Voltage traces in response to a 2-s depolarizing pulse before (**E**) and after veratridine (**F**). **G, H:** Quantification of bistability through ΔV and ΔI. ΔV and ΔI represent the range of holding potentials and holding currents, respectively, over which self-sustained firing can be observed. Each is defined as the difference between the most depolarized and the most hyperpolarized value (potential for ΔV, current for ΔI) at which self-sustained firing is triggered or maintained (see Methods). Paired data from individual motoneurons (n = 8) are linked and overlaid on violin and box-and-whisker plots. *P < 0.05, **P < 0.01, two-tailed Wilcoxon signed-rank test.

Patch-clamp recordings were performed on lumbar motoneurons before and after application of veratridine, a selective enhancer of $I_{NaP}$, to test our model predictions. As in our previous apamin experiments, we measured the amplitude, duration and area of the sADP induced by a short depolarizing current pulse. Veratridine application resulted in a significant increase in the amplitude and area of the sADP, while its duration was markedly reduced (Fig. 7A-D). This pattern indicates a more powerful but shorter depolarizing response in the presence of veratridine. In addition to the modification of sADP properties, veratridine increased the likelihood for motoneurons to display bistable behavior. Under the effect of veratridine, motoneurons were capable of developing plateau potentials at more hyperpolarized membrane potentials, as evidenced by a significant shift in Vh min from −56.9 mV to −61.6 mV (p < 0.01; Wilcoxon matched pairs test). This was accompanied by significant increases in both the voltage range (ΔV) and current range (ΔI) over which bistability could be observed (Fig. 7E-G). These results suggest that veratridine extends the range of membrane potentials and injected currents supporting bistability.

Taken together, these findings support our computational predictions that increasing $I_{NaP}$ with veratridine facilitates $I_{CAN}$-driven bistability by enhancing depolarization (Fig. 6). Our theoretical and experimental results highlight the synergistic interaction between $I_{CAN}$ and $I_{NaP}$ in modulating motoneuron excitability and provide mechanistic insight into the generation of bistable firing patterns.

### Bistability based on $I_{NaP}$ and the role of $K_{\;\;out}^{+}$

For $I_{NaP}$-based bistability to be expressed, the $I_{NaP}$ current must be active when spiking takes place. This sustained activation is necessary to provide the depolarization required for the maintenance of spiking.

**Figure 8.**
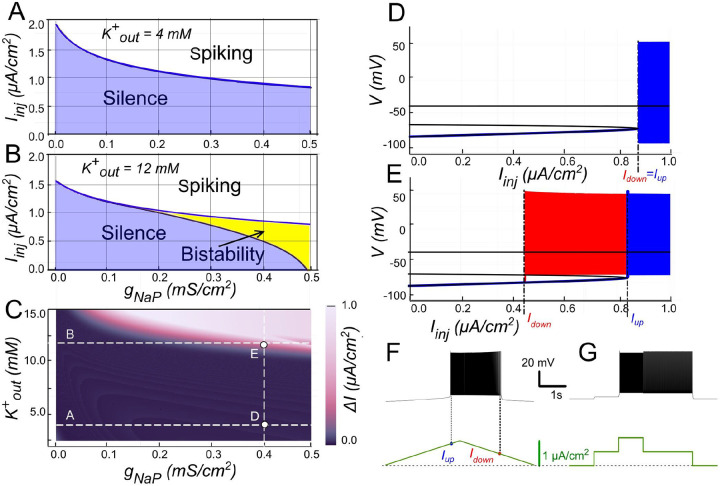
Bistability based on $I_{NaP}$ and the role of $K_{out}$. **A**. Activity regimes of the model neuron depending on the injected current $\left(I_{inj}\right)$ and the conductance of persistent sodium current $\left(g_{NaP}\right)$ at baseline $K^{+}$ extracellular concentration $({K^{+}}_{out})=4\mathrm{mM}$. At all values of $g_{NaP}$ as the injected current changes, the model transitions from silence to spiking and back with no hysteresis which indicates no bistability. **B**. Activity patterns of the model neuron depending on the injected current and $g_{NaP}$ conductance at $({K^{+}}_{out})=12\;\mathrm{mM}$. Once $g_{NaP}$ exceeds approximately 0.15 mS/cm^2^, bistability emerges. **C**. Adjusting the sodium persistent inward current $\left(I_{NaP}\right)$ conductance $\left(g_{NaP}\right)$ and extracellular potassium concentration $\left({K^{+}}_{out}\right)$ in a model neuron reveals bistable regimes. The range of injected current where bistability occurs is shown in color, with black indicating no bistability. Higher ${K^{+}}_{out}$ levels require smaller $g_{NaP}$ for bistability, suggesting that increased ${K^{+}}_{out}$ can induce bistability in neurons with otherwise insufficient $g_{NaP}$ expression. **D**. at $g_{NaP}=0.4\;mS∕cm^{2}$ and $({K^{+}}_{out})=4\;\mathrm{mM}$ no bistability is observed. **E**. However, as ${K^{+}}_{out\;t}$ is increased to 12 mM, bistability emerges, as illustrated by ramp (**F**) and step current protocols (**G**).

When $g_{CAN}$ was set to zero, $I_{NaP}$ by itself could not support bistability in the parameter range examined (Fig. 8A). The bifurcation diagram in Fig. 8D illustrates why $I_{NaP}$ alone cannot generate bistability. At low injected current $\left(I_{inj}\right)$ (below $\sim 0.85\;\mu A∕{\text{cm}}^{2}$ in Fig. 6D), the only stable state of the system is a low-voltage resting state (stable node). For values of $I_{inj}$ above this threshold, the stable node merges with the saddle and then ceases to exist. At this point the system transitions to a stable limit cycle, representing a spiking regime. Of special interest is that when spiking the membrane hyperpolarizes to a level below the node after each action potential, indicating a transition through the saddle-node-on-invariant-circle (SNIC) bifurcation. With a reduction of the input current, a return to the silent regime occurs via the same bifurcation. The large hyperpolarization between action potentials causes complete deactivation of $I_{NaP}$, accounting for its inability to support spiking following a reduction of the injected current.

The post-spike hyperpolarization seen in the model is mainly due to the activation of a potassium rectifier current $\left(I_{Kdr}\right)$. This hyperpolarization appears of sufficient magnitude to inactivate $I_{NaP}$. We can modulate $I_{Kdr}$ by modifying $K_{\;\;out}^{+}$. A higher $K_{\;\;out}^{+}$ leads to a more depolarized potassium reversal potential. This effectively reduces $I_{Kdr}$, and consequently, the after-spike hyperpolarization it causes. This reduction is large enough to make bistability possible, as shown in the bifurcation diagrams in Figs. 8B, E.

In the scenario shown in Fig. 8E, when the injected current crosses a value $0.8\;\mu A∕{\text{cm}}^{2}$, the resting state is eliminated through the same saddle-node bifurcation. However, instead of generating a new limit cycle, the system transitions to a pre-existing one without post-spike hyperpolarization. When the injected current is reduced, the limit cycle persists until the injected current reaches approximately $0.45\;\mu A∕{\text{cm}}^{2}$. At this point, the limit cycle intersects with the saddle point and disappears through a saddle-loop (homoclinic) bifurcation, causing the system to return to the resting state. When the injected current values fall between 0.45 and $0.85\;\mu A∕{\text{cm}}^{2}$ both the resting state and the spiking regime coexist, indicating that the system is bistable. A key characteristic of this type of bistability is that the voltage in the resting state is lower than the voltage range of the limit cycle.

The presence of bistable behavior, where both silence and spiking behaviors are possible, is dependent on the value of the $I_{NaP}$ conductance, $g_{NaP}$, and the injected current values. At $K_{\;\;out}^{+}=12\;\text{mM}$, this bistable behavior emerges if $g_{NaP}$ exceeds 0.1-0.2 mS/cm^2^ (see Fig. 8B). The range of injected current values exhibiting bistability initially starts narrow and widens as $g_{NaP}$ increases.

To understand the relationship between $K_{\;\;out}^{+}$ and the presence of bistability, we varied both $K_{\;\;\text{out}}^{+}$ and $g_{NaP}$, and determined the range of bistability in terms of the injected current (0 indicating no bistability). As illustrated in Fig. 8C, for $g_{NaP}$ values below 0.5 mS/cm^2^ bistability occurs when $K_{\;\;out}^{+}$ surpasses 10 mM, and the required $g_{NaP}$ value decreases as $K_{\;\;out}^{+}$ increases. For instance, in a neuron with a $g_{NaP}$ of 0.25 mS/cm^2^ bistability can be triggered by raising the extracellular potassium concentration $\left(K_{\;\;out}^{+}\right)$ above ~12 mM.

### The role of slowly inactivating potassium current $\left(I_{Kv1.2}\right)$

Recent work indicates that the potassium current $I_{Kv1.2}$ is prevalent in bistable motoneurons (Harris-Warrick et al., 2024). Its direct role in the production of bistable behaviour has not been completely revealed yet. Kv1.2 channels inactivate slowly after repetitive neuronal firing; such slow inactivation would in theory lead to a positive feedback loop: as $I_{Kv1.2}$ becomes less active during ongoing spiking, the cell remains more excitable and can persist in an active state. On the other hand, when the neuron is silent, Kv1.2 channels are fully available, promoting membrane hyperpolarization and suppressing firing.

To dissect the true contribution of $I_{Kv1.2}$, we used computational models to investigate the dynamics of transitions between quiescent and firing states, manipulating both the injected current and $I_{Kv1.2}$. Noteworthy, within our simulations, $I_{Kv1.2}$ was not capable of attaining the real bistability. Instead, the model neurons displayed patterns of periodic bursting, alternating between spiking and quiescence, but over an extremely limited range of input currents.

**Figure 9.**
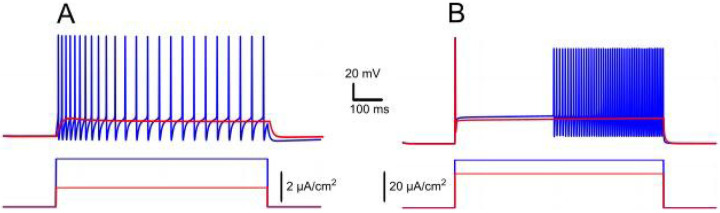
Ramp/delayed excitation versus spike frequency adaptation in response to rectangular current injection. **A**. Model response with $I_{KCa}$ alone ($g_{Kv1.2}=0$, $g_{KCa}=1\;mS∕cm^{2}$, $g_{NaP}=g_{CAN}=0$), displaying spike frequency adaptation as $I_{KCa}$ activation hyperpolarizes the neuron, reducing firing rate over time. **B**. Model response with $I_{Kv1.2}$ alone ($g_{Kv1.2}=2\;mS∕cm^{2}$, $g_{KCa}=0$, $g_{NaP}=g_{CAN}=0$), showing delayed excitation and a ramping firing rate due to slow inactivation of $I_{Kv1.2}$. The membrane potential exhibits an initial jump followed by gradual depolarization, leading to progressively increasing spike frequency.

The signature of $I_{Kv1.2}$ current is a delayed excitation with a ramping firing rate in response to a rectangular injected current stimulus (Bos et al., 2018). To isolate and understand the effects of $I_{Kv1.2}$ alone, we set both $G_{NaP}$ and $G_{CAN}$ to zero in our model. Figure 9 illustrates the response of a model neuron expressing $I_{Kv1.2}$ to a rectangular current stimulus in comparison with the response of the neuron expressing $I_{KCa}$.

In the presence of $I_{Kv1.2}$, upon application of a depolarizing current, the membrane potential exhibits an initial jump followed by a slow depolarization (Fig. 9B). If the stimulus is sufficiently strong, this leads to the neuron beginning to fire with a progressively increasing frequency. The slow depolarization and the ramping spike frequency are due to the very slow kinetics of $I_{Kv1.2}$. Initially, depolarization rapidly activates this current, which temporarily suppresses firing.

However, this same depolarization initiates a slow inactivation process where the inactivation gating variable gradually decreases, reducing the current and allowing for further, albeit slow, depolarization. The time constant for the inactivation gating variable of $I_{Kv1.2}$ is approximately 2.5 s (see Methods), which governs the rate of this process. Depending on the stimulus intensity, this slow depolarization can push the neuron's membrane potential beyond the spiking threshold, initiating firing. As $I_{Kv1.2}$ continues to inactivate, the firing rate gradually increases.

In contrast, the model without $I_{Kv1.2}$ exhibits spike frequency adaptation, which is mediated by $I_{KCa}$ (Fig. 9A; the effects of $I_{KCa}$ are detailed in the “Interplay between $I_{CAN}$ and $I_{KCa}$ and ADP versus AHP” subsection). Here, the firing rate decreases over time due to the activation of $I_{KCa}$ current which hyperpolarizes the neuron.

When both currents ($I_{Kv1.2}$ and $I_{KCa}$) are present in the model, the response – whether it shows ramping or adaptation – depends on the balance between these two currents. This interaction can lead to complex dynamics where the slow inactivation of $I_{Kv1.2}$ competes with the calcium-dependent potassium current’s tendency to stabilize or reduce firing frequency.

## Discussion

In this study, we present a single-compartment computational model of motoneurons to dissect the ionic mechanisms underlying bistability. Our simulations demonstrated that bistable firing can emerge robustly through the synergistic interactions among $I_{CaL}$, CICR, and $I_{CAN}$, without the necessity of explicit dendritic morphology. We further showed that bistability is strongly modulated by opposing currents such as the $I_{KCa}$ and facilitated by $I_{NaP}$. These findings offer new insights into the spatial and molecular dynamics regulating motoneuron bistability.

### Motoneuron bistability critically depends on the $I_{CaL}$-CICR-$I_{CAN}$ loop

Early studies identified persistent inward currents in motoneurons as key drivers of bistability, attributing sustained firing to L-type calcium currents (Schwindt & Crill, 1980; Hounsgaard, Hultborn, et al., 1988; Hounsgaard, Kiehn, et al., 1988; Hounsgaard & Mintz, 1988; Hounsgaard & Kiehn, 1989; Hounsgaard & Kjaerulff, 1992; Svirskis & Hounsgaard, 1997). These currents were believed to be responsible for the generation and the maintenance of the plateau potentials, and the conclusion was also supported by the dendritic distribution of Cav1.3 channels, low-threshold, L-type channels subtype (Simon et al., 2003; Zhang et al., 2008). This concept was strengthened by computational models, as it was frequently necessary to model dendritic $I_{CaL}$ to reproduce experimental plateaux properties (Booth & Rinzel, 1995; Booth et al., 1997; Carlin et al., 2000; ElBasiouny et al., 2005; Bui et al., 2006; Carlin et al., 2009; Kim & Jones, 2011). However, recent experimental evidence has shifted this view, suggesting that $I_{CaL}$ primarily acts as a trigger, while other currents, notably the $I_{CAN}$, play a central role in sustaining bistability (Bos et al., 2021).

Our computational findings reinforce and extend this emerging view. First, the model obviously shows that the initial Ca^2+^ entry through $I_{CaL}$ is insufficient to sustain bistability because of the quick removal of Ca^2+^ from the cytosol. Instead, CICR is an important amplification, producing a positive feedback loop involving $I_{CaL}$-triggered Ca^2+^ influx, CICR-mediated Ca^2+^ release from intracellular stores, subsequent activation of $I_{CAN}$, and further depolarization to sustain prolonged firing states. Accordingly, a major functional implication of our model is that CICR surpasses the capacity of Ca^2+^ pumps to rapidly clear cytoplasmic Ca^2+^, and therefore enables sustained intracellular Ca^2+^, long-lasting $I_{CAN}$ activation and bistability. This prediction finds support in experimental findings CICR blockade abolishes motoneuron plateau potentials despite the presence of $I_{CaL}$ currents (Bouhadfane et al., 2013; Bos et al., 2021; Harris-Warrick et al., 2024). Together, the results emphasize a functional triad, $I_{CaL}$, CICR, and $I_{CAN}$, contributing to motoneuron bistability.

These findings raise the possibility that this triad could be a generic feed-forward principle for plateau generation in a diverse range of neuronal populations, with implications for motor control, sensory processing, and even memory formation. For instance, $I_{CAN}$ drives NMDA-dependent plateaus in lamprey reticulospinal neurons to initiate locomotion (Di Prisco et al., 2000) and is involved in inspiratory bursting in the preBötzinger complex (Jasinski et al., 2013; Toporikova & Butera, 2011). In a similar manner, $I_{CAN}$ contributed to the generation of sensory-related plateaus in dorsal horn neurons, whereas in hippocampal and cerebral neurons $I_{CAN}$ is involved in the generation of memory-linked plateaus (Fraser & MacVicar, 1996; Morisset & Nagy, 1999; Yan et al., 2009).

### Suppression of $I_{CAN}$-based bistability by $I_{KCa}$

The model also introduces a critical regulatory dimension to motoneuron bistability by highlighting the interplay between $I_{CAN}$ and $I_{KCa}$. Both currents are activated by intracellular $Ca^{2+}$, yet they have opposing effects: $I_{KCa}$ induces hyperpolarization producing an afterhyperpolarization (AHP), while $I_{CAN}$ drives depolarization. Our model demonstrates that when $I_{KCa}$ predominates, it acts as a brake on $I_{CAN}$-driven plateau potentials, preventing their onset. Experimentally, pharmacological reduction of $I_{KCa}$ by apamin unmasks latent plateau potentials, confirming the validity of our model predictions (Hounsgaard & Mintz, 1988; Hounsgaard & Kiehn, 1993).

This interplay between $I_{CAN}$ and $I_{KCa}$ may shape motoneuron diversity and function. Recent evidence shows that bistability is largely restricted to larger motoneurons (Harris-Warrick et al., 2024), potentially due to differences in the $I_{CAN}∕I_{KCa}$ ratio. Larger motoneurons exhibit stronger $I_{CAN}$ currents and may also express less $I_{KCa}$, a pattern that can 6be supported by size-dependent differential expression of SK channels (Deardorff et al., 2013). This reduced hyperpolarizing drive, combined with enhanced $I_{CAN}$, likely promotes plateau potentials and bistable firing. Together, these findings highlight how intrinsic ion channel diversity may underlie motoneuron functional heterogeneity and their variable propensity for bistability.

Our findings also identify $K_{\;\;out}^{+}$ concentration as a critical factor influencing $I_{KCa}$ efficacy. Our simulations show that elevating $K_{\;\;out}^{+}$ reduces $E_{K}$ negativity, weakening $I_{KCa}$ even if $G_{KCa}$ does not change, and shifting the balance towards $I_{CAN}$, thereby enhancing bistability (Fig. 4). This mechanism may have profound implications in pathological conditions, such as spinal cord injury (SCI), where astrocytic Kir4.1 alteration impairs K^+^ buffering (Olsen et al., 2010; Benson et al., 2023; Barbay et al., 2024), likely leading to elevated $K_{\;\;out}^{+}$, a phenomenon observed in epilepsy (Djukic et al., 2007; Tong et al., 2014). This mechanism provides a plausible pathophysiological basis for amplifying $I_{CAN}$-driven bistability, promoting self-sustained firing and contributing to spasticity (Bennett et al., 2001; C. Brocard et al., 2016).

### Facilitation of $I_{CAN}$-mediated bistability by $I_{NaP}$

While $I_{CAN}$ is the main driver for the bistability, $I_{NaP}$ also acts as an essential modulator, expanding the regime where bistability emerges. Our simulations reveal that increasing $I_{NaP}$ conductance lowers the threshold for $I_{CAN}$-mediated bistability, enabling sustained firing even when $I_{CAN}$ alone is insufficient (Fig. 6). $I_{NaP}$ likely facilitates this effect by enhancing subthreshold depolarization, thereby setting the action potential threshold (Crill, 1996) and enabling repetitive spiking (Kuo et al., 2006), a prerequisite for activating the $I_{CaL}$-CICR-$I_{CAN}$ triad. Consistent with this mechanism, the pharmacological block of $I_{NaP}$ by riluzole, (an established inhibitor of $I_{NaP}$), reliably suppresses self-sustained firing in bistable motoneurons (Bouhadfane et al., 2013; Drouillas et al., 2023). At the first sight, the ability of Riluzole to eliminate bistability may appear to support that plateau potentials are inherently dependent on $I_{NaP}$. Our current results as well as previous experimental data, however, suggest that such interpretation is likely to be misleading. Indeed, the persistence of plateau potentials, observed as prolonged sADP following brief stimuli in presence of TTX (Bouhadfane et al., 2013; Drouillas et al., 2023), evidenced that $I_{NaP}$ facilitates but can not by itself generate plateau potentials. Instead, $I_{CAN}$ remains the essential substrate for maintaining plateau potentials, and $I_{NaP}$ acts to lower the activation threshold and promote the conditions (repetitive spiking) necessary for bistability to be manifest. The synergy between $I_{NaP}$ and $I_{CAN}$ is not exclusive of motoneurons; analogous interactions have been reported in the preBotzinger complex, where the system uses both currents together to produce bursting (Jasinski et al., 2013; Phillips et al., 2019, 2022).

Under physiological conditions, it is possible that $I_{NaP}$ alone cannot sustain bistability due to the hyperpolarization produced by potassium currents like $I_{KCa}$ and $I_{Kdr}$, which deactivates $I_{NaP}$ between spikes (Fig. 8). We show that elevating $K_{\;\;out}^{+}$ mitigates this hyperpolarization by depolarizing $E_{K}$, allowing $I_{NaP}$ to contribute to bistability at higher $g_{NaP}$ values (Fig. 8C). This has been clearly demonstrated in our simulations, where a pure $I_{NaP}$-based bistability is observed at $K_{\;\;out}^{+}$ approaching or exceeding 12 mM. Once more, this result might have an impact on pathological states conditions such as SCI, where $I_{NaP}$ is enhanced (Bennett et al., 2001; Y. Li et al., 2004; Harvey et al., 2006; C. Brocard et al., 2016). This increase in $I_{NaP}$ together with high $K_{\;\;out}^{+}$, may exacerbate bistability leading to spasticity. To this end, the repurposing of Riluzole (Rilutek©), initially used for ALS treatment, is being explored to target spasticity in SCI patients (Cotinat et al., 2023).

### The role of slowly inactivating potassium current $\left(I_{Kv1.2}\right)$

The slowly inactivating potassium current viaKv1.2 channels $\left(I_{Kv1.2}\right)$, is an important modulator of motoneuron discharge dynamics, significantly driving delayed excitation and the recruitment of a ramping discharge rate (Bos et al., 2018). This current is size-dependent in motoneurons, larger neurons having higher values (Harris-Warrick et al., 2024). The slow inactivation rate of Kv1.2 channels means that their outward current gradually decreases during constant depolarization, leading to ramp-like depolarizing potential and firing acceleration once the firing threshold is reached.

However, despite its influence on firing patterns, our computational modeling showed that $I_{Kv1.2}$ is not necessarily enough to produce classical bistability. When $I_{Kv1.2}$ was the only current altered in the model, it produced periodic bursting rather than exhibiting a robust switching between quiescent and persistently firing states, and this behavior only extended over a very narrow range of inputs.

In summary, $I_{Kv1.2}$ is essential for delayed excitation and ramping firing in motoneurons with its expression correlating with bistability in larger cells. However, it primarily acts as a modulator of firing dynamics, while robust bistable behavior appears to depend on the interplay of other persistent inward currents like the $I_{CaL}$-CICR-$I_{CAN}$ triad.

### Serotonin and bistability

Serotonin powerfully shapes plateau potentials and self-sustained firing of motoneurons in the spinal cord (Heckman et al., 2008). In decerebrate cats it has been demonstrated that bistability necessitates brainstem monoamines (Conway et al., 1988; Hounsgaard, Hultborn, et al., 1988; Lee & Heckman, 1998b, 1999). In particular, acute spinalization removes supraspinal monoaminergic inputs which mitigates bistable properties of motoneurons, but serotonin reintroduction restores plateau potentials (Hounsgaard, Hultborn, et al., 1988). This effect is consistent across species since adding serotonin *in vitro* to the turtle spinal cord enhances excitability and bistability (Hounsgaard & Kiehn, 1985, 1989).

Mechanistically, serotonin acts primarily through 5-HT2 receptors, amplifying predominant ionic currents that promote bistability. First, it increases dendritic L-type calcium currents (Hounsgaard & Kiehn, 1989; Perrier & Hounsgaard, 2003; Perrier & Delgado-Lezama, 2005; Perrier & Cotel, 2008) that, in our model, mediate calcium build-up and $I_{CAN}$ activation to sustain plateau potentials. Second, serotonin shifts $I_{NaP}$ activation toward more hyperpolarized potentials, thereby amplifying neuronal excitability (Y. Li & Bennett, 2003; Harvey et al., 2006). This effect is in accord with a previous study showing the facilitatory effect of $I_{NaP}$ in the generation of $I_{CAN}$-mediated bistability. Third, serotonin inhibits outward currents, specifically $I_{KCa}$ mediating AHP, promoting high-frequency firing (Grunnet et al., 2004). Because $I_{KCa}$ can be activated by both spike-induced and dendritic Ca^2+^ influx (X. Li et al., 2007), plateau potentials themselves may trigger $I_{KCa}$, determining their duration. Blocking $I_{KCa}$ with apamin enhances bistability in turtle motoneurons (Hounsgaard, Kiehn, et al., 1988; Hounsgaard & Kiehn, 1993). As predicted by our model, this happens because the reduction $I_{KCa}$ discloses $I_{CAN}$-driven bistability by limiting hyperpolarization. Together, these modulatory actions significantly reshape motoneuron input-output properties, favoring sustained excitability and persistent firing.

The involvement of serotonin becomes much more striking in a pathological context like SCI, not due to its direct action, but because of its sudden absence after the loss of descending inputs. This depletion initially leads to hypofunction of motoneurons, otherwise known as “spinal shock” (Schadt & Barnes, 1980), mirroring the loss of bistability in our model upon blockade of $I_{CAN}$ or CICR. However, with time motoneurons gradually restore excitability and plateau potentials, a phenomenon clinically linked to the emergence of spasticity and hyperreflexia in chronic injury phases (Bennett et al., 2001). This recovery is driven by plastic changes in serotonin receptor function, particularly the emergence of constitutively active 5-HT_2B_ and 5-HT_2C_ receptors, which restore persistent inward currents and plateau firing even without serotonergic input (Y. Li & Bennett, 2003; Murray et al., 2010, 2011; D’Amico et al., 2013; Tysseling et al., 2017). This aligns with our findings that increased $I_{NaP}$ enhances $I_{CAN}$-driven bistability, contributing to spasticity and hyperreflexia.

### Limitations and future directions

While our single-compartment computational model successfully reproduces essential properties of motoneuron bistability, we acknowledge that its simplified architecture limits the representation of spatial complexity of motoneurons. By collapsing dendrites into a single compartment, our model contrasts from multi-compartment paradigms, which explicitly emphasize dendritic persistent inward currents as pivotal players in the mechanism of bistability (Booth et al., 1997; Carlin et al., 2000; ElBasiouny et al., 2005; Bui et al., 2006). Although our findings suggest that somatic mechanisms can suffice, dendritic CICR and $I_{CaL}$ localization might play a significant role to amplify calcium signaling.

Future studies may use multi-compartment models to explore spatial dynamics of $I_{CaL}$, CICR, and $I_{CAN}$, potentially revealing region-specific contributions to bistability. Such detailed models could help clarify whether dendritic versus somatic localization significantly alters the conditions and robustness of bistability, and elucidate whether dendritic compartments play an essential amplifying role beyond the capabilities represented by our current simplified model. In this context, resolving the precise localization of $I_{CAN}$ channels is critical; however, progress is currently impeded by the absence of highly specific antibodies against TRPM5, the presumed molecular correlate. Furthermore, although our model focuses primarily on L-type calcium channels, motoneurons express T-type and N-type calcium channels as well (Umemiya & Berger, 1995; Hivert et al., 1995; Viana et al., 1997). It is thus possible that other calcium currents, in addition to L-type, could underlie activation of the plateau (see (Bouhadfane et al., 2013)). Future modeling efforts should incorporate these additional channel types to assess their cooperative or redundant roles in motoneuron bistability.

In summary, while our minimal model indicates that explicit implementation of dendritic morphology is not strictly necessary to reproduce bistable firing, future models incorporating anatomically-detailed dendritic compartments will be essential to fully resolve the spatial origins of the underlying ionic mechanisms.
